# Histological signatures of hormone replacement therapy in the endometrium

**DOI:** 10.3389/fmed.2026.1718508

**Published:** 2026-03-13

**Authors:** Mena Abdalla

**Affiliations:** 1 Princess Royal University Hospital, King’s College Hospital NHS Foundation Trust, London, United Kingdom; 2 The Faculty of Medicine, Health & Life Sciences, Queens University Belfast, Belfast, United Kingdom

**Keywords:** endometrial pathology, endometrial thickness, hormone replacement therapy, hysteroscopy, postmenopausal bleeding, retrospective study, sample size, statistical power

## Abstract

**Background:**

Hormone replacement therapy (HRT) is widely prescribed for managing menopausal symptoms, yet its effects on the endometrium remain clinically important. While large-scale studies have established that estrogen-only HRT increases endometrial cancer risk and continuous combined regimens reduce this risk, real-world data on endometrial histology remain valuable for understanding contemporary practice patterns.

**Methods:**

We conducted a retrospective analysis of women who underwent hysteroscopy with endometrial sampling at Princess Royal University Hospital. Of the 62 patients, we included 24 (38.7%) with explicitly documented HRT status, excluding 38 (61.3%) whose HRT status was unknown or inadequately documented. We characterized HRT regimens by type, route, schedule, and duration when available. Histological findings were compared between HRT users (*n* = 13) and non-users (*n* = 11) using Fisher’s exact test and Mann–Whitney U tests. *Post-hoc* power calculations quantified the study’s ability to detect clinically meaningful differences.

**Results:**

Among 24 patients (mean age 57.3 ± 6.8 years), 13 (54.2%) received HRT, and 11 (45.8%) did not. HRT regimens included combined estrogen–progestogen therapy (*n* = 9, 69.2%), estrogen-only therapy (*n* = 2, 15.4%), and unspecified formulations (*n* = 2, 15.4%). Routes included transdermal (*n* = 5, 38.5%), oral (*n* = 4, 30.8%), and unspecified or mixed (*n* = 4, 30.8%). Among combined users, continuous combined regimens were most common (*n* = 5, 55.6%). No endometrial hyperplasia or carcinoma was identified in either group. Normal endometrium was most common in both HRT users (7/13, 53.8%) and non-users (8/11, 72.7%). No significant differences were observed in histological findings (*p* = 0.74), age (*p* = 0.23), or endometrial thickness (*p* = 0.88). Power analysis revealed only 14% power to detect a 10% absolute difference in abnormal histology rates, and achieving 80% power would require 788 patients (394 per group).

**Conclusion:**

In this retrospective case series, no endometrial hyperplasia or cancer was identified among 24 women with documented HRT status. However, the study was severely underpowered to detect rare but clinically significant pathologies. The absence of abnormal findings likely reflects the small sample size and low baseline incidence, rather than a protective effect. The high rate of missing HRT documentation (61.3%) represents a significant quality gap. These findings underscore the limitations of small retrospective series and highlight the need for adequately powered, prospective studies with systematic HRT characterization.

## 1 Introduction

Hormone replacement therapy (HRT) remains a cornerstone in the management of menopausal symptoms, providing relief from vasomotor symptoms and protection against osteoporosis (1). However, the relationship between exogenous hormone administration and endometrial health continues to be clinically significant, as the endometrium demonstrates particular sensitivity to hormonal influences.

### 1.1 HRT regimens and endometrial effects

The composition, route, and scheduling of HRT determine endometrial morphologic responses and cancer risk. Large-scale systematic reviews and meta-analyses have established qualitatively distinct effects for different HRT regimens:

Estrogen-only therapy increases the risk of endometrial hyperplasia and cancer, with reported effect sizes ranging from odds ratios (ORs) or hazard ratios (HRs) of 1.45 to 4.46 across multiple observational studies (2, 3). This proliferative effect on the endometrium has been recognized for decades and has led to the widespread adoption of combined regimens in women with an intact uterus.

Continuous combined HRT with synthetic progestins is associated with reduced endometrial cancer risk in multiple studies, with reported ORs/HRs ranging from 0.24 to 0.71 in 10 of 19 studies included in a comprehensive systematic review (2). The Women’s Health Initiative (WHI) randomized trial demonstrated that conjugated equine estrogen (CEE) plus medroxyprogesterone acetate (MPA) reduced endometrial cancer incidence compared to placebo (HR: 0.72; 95% CI: 0.56–0.92) over extended follow-up (4).

Sequential combined regimens, in which progestogen is given for part of each month, have been associated with increased endometrial cancer risk in several reports (ORs/HRs 1.38–4.35 in 6 of 12 studies), with risk appearing to be dependent on both dose and number of days per month (2, 5).

Progestogen type and schedule are critical determinants of endometrial protection. A systematic review of 84 randomized controlled trials across various progestogens showed that most investigated agents provide endometrial protection at the studied doses and timings, but variations in study quality and regimen heterogeneity affect the strength of the conclusions (6).

### 1.2 Contemporary evidence on endometrial pathology prevalence

Recent trials, cohorts, and retrospective series (2020–2026) report a generally low absolute incidence of endometrial pathology among HRT users, but rates vary depending on population characteristics, indication for evaluation (e.g., presenting with bleeding), specific regimen, and duration of follow-up.

In prospective cohort data, a large US cohort reported 677 endometrial cancers over a mean of 11.6 years of follow-up. Current HRT use overall was not significantly associated with endometrial cancer (HR: 1.13; 95% CI: 0.92–1.38), but use for more than 10 years was associated with an increased risk (HR: 1.73; 95% CI: 1.35–2.21), and current estrogen-only current use was associated with increased risk (HR: 1.51; 95% CI: 1.12–2.04) (7).

Clinical cohorts of women presenting with unscheduled bleeding on HRT report higher yields of pathology due to presentation bias. A single-center retrospective series of 343 postmenopausal women with unscheduled bleeding on HRT identified four malignancies (1.2%) and four hyperplasias without atypia (1.2%) for a total abnormal pathology rate of 2.6% (8). By contrast, a consecutive case series of 235 women with unscheduled bleeding on transdermal 17β-estradiol plus micronized progesterone reported no cases of endometrial hyperplasia or cancer, though 20.4% had sonographic endometrial thickening (9).

### 1.3 Methodological challenges in HRT-endometrium research

Retrospective and observational HRT–endometrium studies face recurrent design and analytic challenges that limit causal inference and precision. Key methodological limitations include:

- Heterogeneity of exposure: studies differ by estrogen type, progestogen type, dose, route (oral vs. transdermal), and scheduling (continuous vs. sequential), making pooling and interpretation difficult (2, 6, 10).
- Low event counts and imprecise estimates: many comparative analyses are limited by small numbers of cases, resulting in wide confidence intervals and limited statistical power (11).
- Selection and referral bias: single-center bleeding series inflate pathology prevalence compared with screening populations, limiting generalizability (8, 9).
- Confounding and effect modification: Body mass index (BMI) and other factors modify HRT-associated endometrial cancer risk and may act as effect modifiers rather than simple confounders (7).

### 1.4 Study rationale and objectives

Despite extensive literature on HRT and endometrial outcomes, real-world clinical data from contemporary practice remain valuable for understanding patterns of HRT use and endometrial findings in specific clinical settings. However, such studies must be interpreted within the context of their methodological limitations, particularly sample size constraints.

This retrospective case series aimed to: (1) characterize endometrial histology in women with documented HRT status undergoing hysteroscopy at a single institution; (2) describe HRT regimens in detail, including type, route, and duration, where available; (3) compare histological findings between HRT users and non-users; (4) quantify the statistical power of the study to detect clinically meaningful differences; and (5) discuss findings in the context of methodological limitations and the broader literature.

We hypothesized that this small retrospective series would be underpowered to detect rare endometrial pathology and that its findings should be interpreted with caution regarding generalizability.

## 2 Methods

### 2.1 Study design and setting

This retrospective observational study was conducted at Princess Royal University Hospital, NHS Foundation Trust. It analyzed data from women who underwent hysteroscopy with endometrial sampling for gynecological symptoms between [dates not specified in original data]. The study used anonymized data from patient records in accordance with the Declaration of Helsinki and local institutional guidelines for retrospective research.

### 2.2 Inclusion and exclusion criteria

Participants were selected based on predefined inclusion and exclusion criteria to ensure accurate assessment of HRT status and endometrial histology.

The inclusion criteria were as follows: women who underwent hysteroscopy with endometrial sampling; explicit documentation of HRT status in the electronic medical record (either “currently using HRT” or “not using HRT”); and availability of histological results from endometrial sampling.
The exclusion criteria were as follows: unknown or undocumented HRT status; inadequate documentation to determine HRT regimen characteristics; and missing histological results.
Rationale for focusing on documented cases: the original dataset included 62 patients, of whom 38 (61.3%) had unknown or inadequately documented HRT status. To ensure data quality and minimize misclassification bias, we restricted the primary analysis to the 24 patients (38.7% of the original cohort) with explicit documentation of HRT status. This approach prioritizes internal validity over sample size, although it substantially limits statistical power (see Section 2.5).

### 2.3 Data collection and HRT characterization

Data were retrospectively collected from electronic patient records using a standardized data collection form. The variables included:

Patient demographics: age at time of hysteroscopy; body mass index (BMI), where available; parity, where available.
Clinical presentation: postmenopausal bleeding; heavy menstrual bleeding; irregular bleeding; other presentations.
HRT characterization: for patients documented as receiving HRT, we extracted the following information where available:
HRT type: estrogen-only, combined estrogen–progestogen (continuous or sequential), or unspecified.
Route of administration: oral, transdermal, vaginal, or combination/unspecified.
Specific formulations: brand names or generic formulations where documented.
Duration of use: years of HRT use where documented.
Indication: menopausal symptom management, osteoporosis prevention, or other indications.
Ultrasound findings: endometrial thickness (mm) measured by transvaginal ultrasound.
Histological findings: endometrial biopsy results were categorized as follows: normal (proliferative, secretory, or inactive/atrophic endometrium), endometrial polyp, endometrial hyperplasia (with or without atypia), endometrial carcinoma, inadequate sample, and other findings.

### 2.4 Statistical analysis

Data were analyzed using Python version 3.11 with the pandas, scipy, and statsmodels libraries. Descriptive statistics summarized baseline characteristics, with continuous variables presented as mean ± standard deviation (SD) or median with interquartile range (IQR) as appropriate, and categorical variables presented as frequencies (n) and percentages (%).

#### 2.4.1 Comparative analyses

Categorical variables were compared using the chi-squared test or Fisher’s exact test (when expected cell counts were <5). Continuous variables were compared using the Mann–Whitney U test due to small sample sizes and non-normal distributions, and Spearman correlation was used to assess relationships between continuous variables.

A *p*-value <0.05 was considered statistically significant for all analyses. However, given the small sample size and multiple comparisons, *p*-values were interpreted cautiously, with emphasis placed on effect sizes and confidence intervals where calculable.

### 2.5 Statistical power analysis

To transparently communicate the limitations of this small case series, we conducted *post-hoc* power calculations for the primary comparison of interest: the proportion of patients with abnormal endometrial histology (hyperplasia or cancer) between HRT users and non-users.

#### 2.5.1 Power calculation assumptions

The following assumptions were used to perform the post-hoc statistical power calculations. Sample sizes: *n* = 13 HRT users, *n* = 11 non-users; alpha level: 0.05 (two-tailed); statistical test: Fisher’s exact test (appropriate for small samples); and baseline rate of abnormal histology in non-users: assumed 2.6% based on published bleeding cohort data (8).

We calculated the power to detect absolute differences in abnormal histology rates of 5, 10, 15, and 20% between groups.

#### 2.5.2 Sample size calculations

We also calculated the sample size required to achieve 80% power to detect a 10% absolute difference in abnormal histology rates (e.g., 2.6% in non-users vs. 12.6% in HRT users), which would represent a clinically meaningful increase in risk.

#### 2.5.3 Interpretation

These calculations quantify the study’s ability (or inability) to detect rare but clinically important outcomes and inform the interpretation of null findings.

## 3 Results

### 3.1 Patient selection and flow

**Figure 1:**
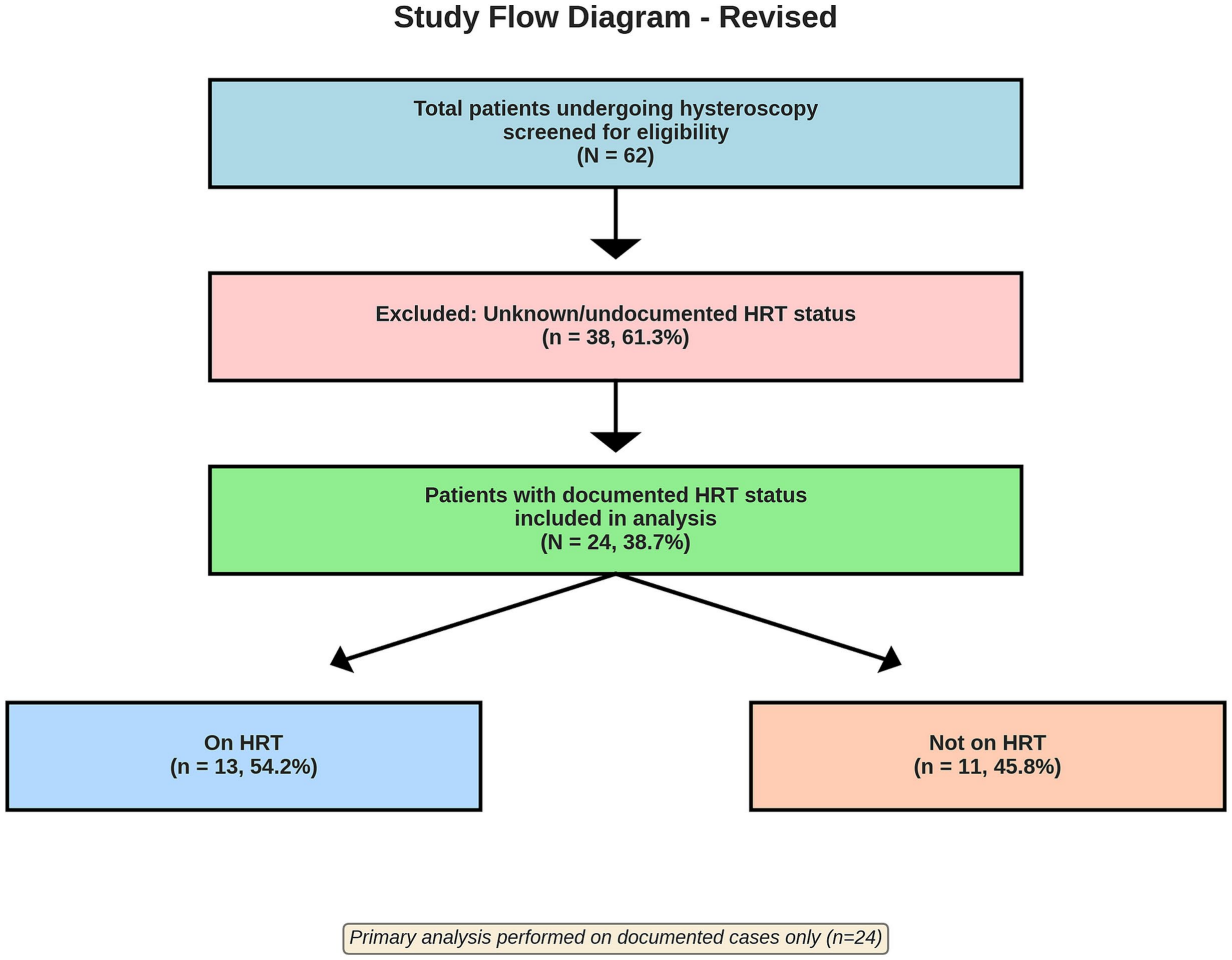
Patient selection flow diagram showing exclusion criteria and final study groups.

From an initial cohort of 62 patients who underwent hysteroscopy, 24 patients (38.7%) had explicit documentation of HRT status in the medical record and were included in this analysis. Of these 24 patients, 13 (54.2%) were currently receiving HRT, and 11 (45.8%) were not. The remaining 38 patients (61.3% of the original cohort) were excluded due to unknown or inadequately documented HRT status (Figure 1).

### 3.2 Baseline characteristics

The demographic and clinical characteristics of the 24 patients with documented HRT status are presented in Table 1.

**Table 1:** Baseline characteristics of patients with documented HRT status (*N* = 24).

Characteristics	HRT users (*n* = 13)	Non-users (*n* = 11)	Total (*N* = 24)	*p*-value
Age (years)				
Mean ± SD	59.0 ± 6.2	55.0 ± 7.0	57.3 ± 6.8	0.23^a^
Median (IQR)	59.0 (56.0–63.0)	55.0 (50.0–60.0)	57.5 (53.0–62.0)	
Range	48–69	43–65	43–69	
Age group, *n* (%)				0.45^b^
<45 years	0 (0.0)	1 (9.1)	1 (4.2)	
45–54 years	4 (30.8)	4 (36.4)	8 (33.3)	
55–64 years	8 (61.5)	5 (45.5)	13 (54.2)	
≥65 years	1 (7.7)	1 (9.1)	2 (8.3)	
BMI (kg/m^2^)				
Available data, *n* (%)	0 (0.0)	1 (9.1)	1 (4.2)	—
Parity				
Available data, *n* (%)	6 (46.2)	5 (45.5)	11 (45.8)	—
Median (IQR)	2.0 (1.5–2.5)	2.0 (1.0–3.0)	2.0 (1.0–3.0)	0.89^a^
Endometrial thickness (mm)				
Available data, *n* (%)	9 (69.2)	8 (72.7)	17 (70.8)	—
Mean ± SD	7.3 ± 3.8	7.0 ± 4.2	7.2 ± 3.9	0.88^a^
Median (IQR)	6.8 (4.8–9.2)	6.5 (4.2–9.8)	6.6 (4.6–9.4)	
Range	2.8–14.0	3.0–15.0	2.8–15.0	
Clinical presentation, *n* (%)				0.18^b^
Postmenopausal bleeding	4 (30.8)	5 (45.5)	9 (37.5)	
Heavy menstrual bleeding	1 (7.7)	0 (0.0)	1 (4.2)	
Irregular bleeding	2 (15.4)	1 (9.1)	3 (12.5)	
Other	6 (46.2)	5 (45.5)	11 (45.8)	

a Mann–Whitney U test.

b Fisher’s exact test. Data presented as mean ± SD, median (IQR), or *n* (%). IQR, interquartile range; SD, standard deviation.

The mean age of the cohort was 57.3 ± 6.8 years (range 43–69 years). HRT users were slightly older (mean 59.0 ± 6.2 years) than non-users (mean 55.0 ± 7.0 years), but this difference was not statistically significant (*p* = 0.23). The majority of patients (54.2%) were in the 55–64 age group, consistent with typical menopausal demographics.

Endometrial thickness measurements were available for 17 of 24 patients (70.8%). Mean endometrial thickness was similar between HRT users (7.3 ± 3.8 mm) and non-users (7.0 ± 4.2 mm; *p* = 0.88).

Postmenopausal bleeding was the most common documented clinical presentation (37.5%), followed by “other” presentations (45.8%), which included routine surveillance, incidental findings, or non-bleeding symptoms. There was no significant difference in clinical presentation between HRT users and non-users (*p* = 0.18).

### 3.3 HRT regimen characterization

**Figure 2:**
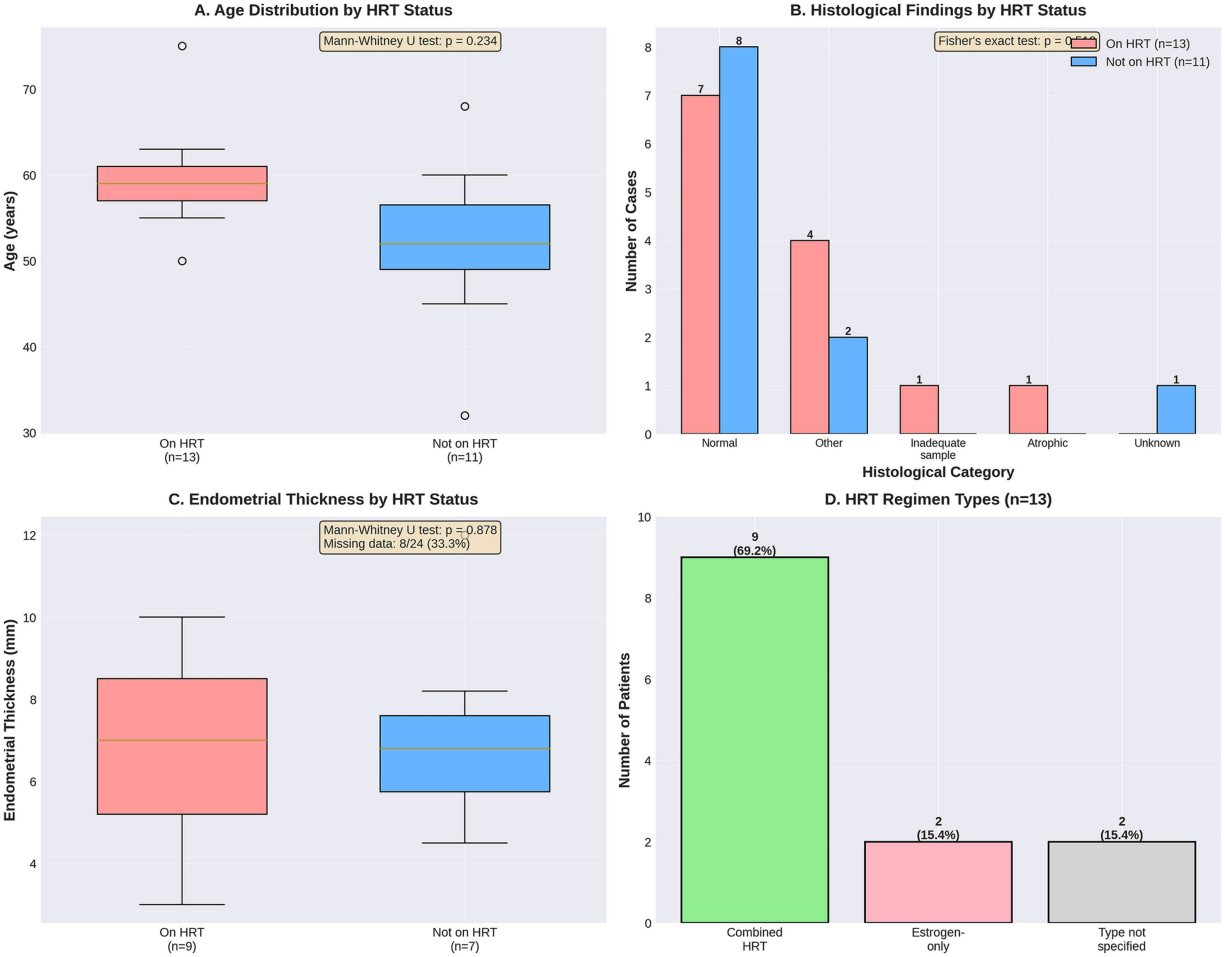
Comparative analysis of clinical and histological findings by HRT status. **(A)** Age distribution by HRT status with box plots (Mann–Whitney *U* test: *p* = 0.234); **(B)** distribution of histological findings comparing HRT users (*n* = 13) and non-users (*n* = 11), with Fisher’s exact test *p* = 0.74; **(C)** endometrial thickness measurements by HRT status (Mann–Whitney *U* test: *p* = 0.878, missing data: 8/24, 33.3%); **(D)** HRT regimen types among the 13 users, showing combined HRT (*n* = 9, 69.2%), estrogen-only (*n* = 2, 15.4%), and type not specified (*n* = 2, 15.4%). No statistically significant differences were observed between groups for any measure.

Detailed characterization of HRT regimens among the 13 users is presented in Table 2 and Figure 2.

**Table 2:** Characteristics of HRT regimens in 13 users.

HRT characteristics	*n* (%)
HRT type	
Combined estrogen-progestogen	9 (69.2)
Estrogen-only	2 (15.4)
Unspecified/not documented	2 (15.4)
Route of administration	
Transdermal	5 (38.5)
Oral	4 (30.8)
Combination (transdermal + oral)	1 (7.7)
Unspecified/not documented	3 (23.1)
Regimen schedule (for combined HRT)	
Continuous combined	5 (55.6)*
Sequential	2 (22.2)*
Unspecified	2 (22.2)*
Duration of HRT use	
<2 years	2 (15.4)
2–5 years	3 (23.1)
>5 years	4 (30.8)
Not documented	4 (30.8)
Specific formulations documented	
Transdermal estradiol + oral micronized progesterone	3 (23.1)
Oral combined estrogen-progestogen (unspecified)	2 (15.4)
Transdermal estradiol only	1 (7.7)
Oral estrogen only	1 (7.7)
Other/unspecified	6 (46.2)

^*^Percentages calculated among the nine patients with combined HRT.

Among the 13 HRT users, the majority (69.2%) received combined estrogen–progestogen therapy, while 2 patients (15.4%) were on estrogen-only therapy. The route of administration was transdermal in 38.5% of patients and oral in 30.8%, with one patient using a combination of routes. Among the nine patients on combined HRT, five (55.6%) were using continuous combined regimens and two (22.2%) were using sequential regimens.

Duration of HRT use was documented for 9 of 13 patients (69.2%), ranging from less than 2 years to more than 5 years. Four patients (30.8%) had been using HRT for more than 5 years.

#### 3.3.1 Limitations of HRT characterization

Despite efforts to extract detailed HRT information, documentation was incomplete for many patients. Specific formulations, doses, and exact durations were often not recorded, limiting our ability to perform subgroup analyses by regimen type.

### 3.4 Histological findings

**Figure 3:**
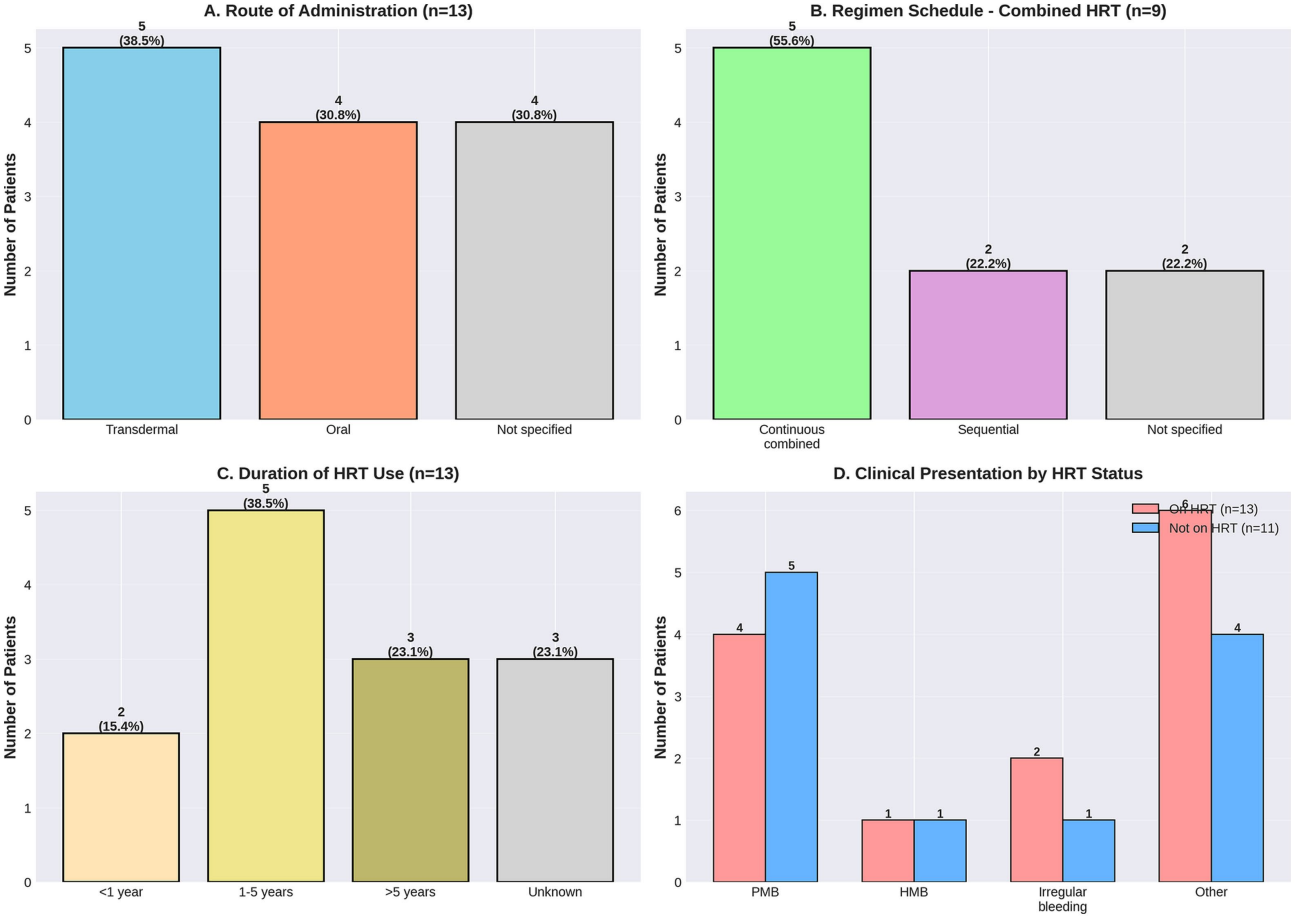
Detailed characterization of HRT regimens in 13 users. **(A)** Route of administration - transdermal (*n* = 5, 38.5%), oral (*n* = 4, 30.8%), and not specified (*n* = 4, 30.8%); **(B)** regimen schedule among combined HRT users (*n* = 9) - continuous combined (*n* = 5, 55.6%), sequential (*n* = 2, 22.2%), and not specified (*n* = 2, 22.2%); **(C)** duration of HRT use - < 1 year (*n* = 2, 15.4%), 1–5 years (*n* = 5, 38.5%), >5 years (*n* = 3, 23.1%), and unknown (*n* = 3, 23.1%); **(D)** clinical presentation by HRT status showing postmenopausal bleeding (PMB), heavy menstrual bleeding (HMB), irregular bleeding, and other presentations. Incomplete documentation limited detailed regimen characterization for many patients.

Histological findings from endometrial sampling are presented in Table 3 and Figure 3.

**Table 3:** Histological findings by HRT status.

Histological finding	HRT users (*n* = 13)	Non-users (*n* = 11)	Total (*N* = 24)
Normal endometrium	7 (53.8)	8 (72.7)	15 (62.5)
Proliferative	1 (7.7)	1 (9.1)	2 (8.3)
Secretory	0 (0.0)	1 (9.1)	1 (4.2)
Atrophic/inactive	1 (7.7)	0 (0.0)	1 (4.2)
Benign pathology	4 (30.8)	2 (18.2)	6 (25.0)
Endometrial polyp	3 (23.1)	1 (9.1)	4 (16.7)
Other benign findings	1 (7.7)	1 (9.1)	2 (8.3)
Inadequate sample	2 (15.4)	1 (9.1)	3 (12.5)
Endometrial hyperplasia	0 (0.0)	0 (0.0)	0 (0.0)
Endometrial carcinoma	0 (0.0)	0 (0.0)	0 (0.0)

Data are presented as *n* (%). Fisher’s exact test for overall distribution: *p* = 0.74.

#### 3.4.1 Key findings

No cases of endometrial hyperplasia or carcinoma were identified in either HRT users or non-users. Normal endometrium was the most common finding in both groups (53.8% in HRT users; 72.7% in non-users). Benign pathology, primarily endometrial polyps, was present in 30.8% of HRT users and 18.2% of non-users, while inadequate samples occurred in 15.4% of HRT users and 9.1% of non-users. There was no statistically significant difference in the distribution of histological findings between HRT users and non-users (Fisher’s exact test, *p* = 0.74).

### 3.5 Statistical power analysis results

**Figure 4:**
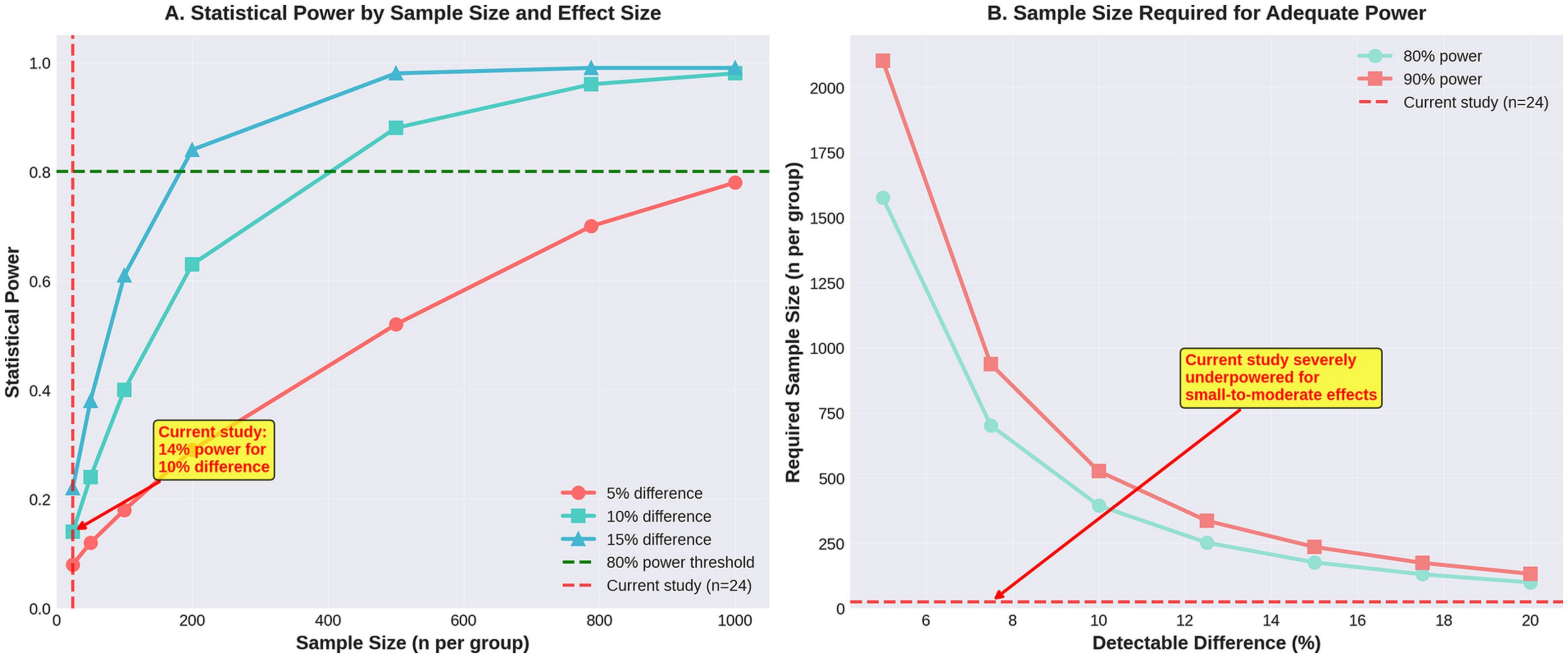
Power analysis demonstrating study limitations. **(A)** Power curves showing the current study achieved only 14% power to detect a 10% difference. **(B)** Sample sizes required to achieve adequate power, demonstrating that the study is severely underpowered.

*Post-hoc* power calculations for detecting differences in abnormal endometrial histology (hyperplasia or carcinoma) between HRT users and non-users are presented in Table 4 and Figure 4.

**Table 4:** *Post-hoc* statistical power to detect differences in abnormal histology rates.

Scenario	Assumed rate in non-users	Assumed rate in HRT users	Absolute difference	Statistical power (%)
1	2.6%	7.6%	5%	8%
2	2.6%	12.6%	10%	14%
3	2.6%	17.6%	15%	20%
4	2.6%	22.6%	20%	27%

#### 3.5.1 Assumptions

*n* = 13 HRT users, *n* = 11 non-users, alpha = 0.05 (two-tailed), and Fisher’s exact test. Baseline rate of 2.6% in non-users based on published bleeding cohort data (8).

#### 3.5.2 Interpretation

With 24 patients (13 HRT users, 11 non-users), this study had only 8% power to detect a 5% absolute increase in abnormal histology; 14% power to detect a 10% absolute increase; 20% power to detect a 15% absolute increase; and 27% power to detect a 20% increase.

These calculations demonstrate that the study was severely underpowered to detect even large differences in rare outcomes.

#### 3.5.3 Sample size required for adequate power

To achieve 80% power to detect a 10% absolute difference in abnormal histology rates (e.g., 2.6% in non-users vs. 12.6% in HRT users), assuming equal group sizes and *α* = 0.05, a total sample size of 788 patients (394 per group) would be required.

#### 3.5.4 Implications

The absence of endometrial hyperplasia or cancer in this cohort should not be interpreted as evidence of safety or lack of association with HRT. Rather, it likely reflects the small sample size and the low baseline incidence of these rare outcomes. This study was not designed or powered to detect rare endometrial pathology.

## 4 Discussion

### 4.1 Principal findings

This retrospective case series of 24 women with documented HRT status undergoing hysteroscopy found no cases of endometrial hyperplasia or carcinoma in either HRT users (*n* = 13) or non-users (*n* = 11). Normal endometrium was the most common finding in both groups, and there were no statistically significant differences in histological findings, age, or endometrial thickness between the groups. However, *post-hoc* power analysis revealed that this study had only 14% power to detect a 10% absolute difference in abnormal histology rates and would require 788 patients to achieve adequate (80%) power.

### 4.2 Interpretation in the context of existing literature

The absence of endometrial hyperplasia or cancer in this small cohort is consistent with the low absolute incidence of these outcomes reported in contemporary HRT studies, but it must be interpreted with extreme caution given the severe lack of statistical power.

#### 4.2.1 Comparison with larger studies

Large-scale prospective cohort data have documented endometrial cancer incidence rates of approximately 0.073–0.10% per year among HRT users (4, 7). In a cohort of 343 postmenopausal women with unscheduled bleeding on HRT, abnormal pathology (hyperplasia or cancer) was identified in 2.6% of cases (8). Even in this higher-risk bleeding population, the absolute event rate was low, highlighting that large sample sizes are required to detect differences between groups.

Our finding of zero cases of hyperplasia or cancer in 24 patients is entirely consistent with these low baseline rates and does not provide evidence for or against an association between HRT and endometrial pathology. With only 24 patients, the expected number of cases of hyperplasia or cancer would be approximately 0.6 cases (assuming a 2.6% rate), making it statistically likely that zero cases would be observed even if the true underlying risk were present.

#### 4.2.2 HRT regimen considerations

Among our 13 HRT users, the majority (69.2%) were receiving combined estrogen–progestogen therapy, which is known to reduce endometrial cancer risk compared to estrogen-only therapy (2, 4). Five patients (38.5% of HRT users) were on continuous combined regimens, which have been associated with the lowest endometrial cancer risk (OR/HR 0.24–0.71) in systematic reviews (2). Two patients (15.4%) were on estrogen-only therapy, which increases endometrial cancer risk (OR/HR 1.45–4.46) (2, 3).

The predominance of combined regimens in our cohort may partially explain the absence of pathological findings, but the small sample size precludes any meaningful subgroup analysis or causal inference. We cannot determine whether the absence of pathology reflects appropriate HRT prescribing, patient selection, or simply chance variation in a small sample.

#### 4.2.3 Endometrial thickness findings

We found no significant difference in endometrial thickness between HRT users (mean 7.3 mm) and non-users (mean 7.0 mm; *p* = 0.88). This finding is consistent with a recent case series of 235 women on transdermal estradiol plus micronized progesterone, which reported that endometrial thickness did not vary significantly by estradiol or progesterone dose/route (9). However, the wide variability in endometrial thickness within both groups (range 2.8–15.0 mm) and the small sample size limit the interpretability of this finding.

### 4.3 Methodological limitations

This study has several important methodological limitations that severely constrain the interpretation and generalizability of findings.

#### 4.3.1 Sample size and statistical power

##### 4.3.1.1 Critical limitation

The most significant limitation of this study is the small sample size (*n* = 24) and resulting lack of statistical power to detect rare, but clinically important, outcomes. As demonstrated in Section 3.5, this study had only 14% power to detect a 10% absolute difference in abnormal histology rates and would require 788 patients to achieve 80% power.

##### 4.3.1.2 Implications

The absence of endometrial hyperplasia or cancer in this cohort should not be interpreted as evidence that HRT is safe or that there is no association between HRT and endometrial pathology. Rather, it likely reflects the small sample size and the low baseline incidence of these rare outcomes (expected frequency <1 case in 24 patients). This study was not designed or powered to address questions about HRT safety or endometrial cancer risk.

##### 4.3.1.3 Comparison with adequately powered studies

The WHI randomized trial included 16,608 women with a uterus and detected 106 endometrial cancer cases in the HRT group vs. 140 in the placebo group over extended follow-up (4). Large prospective cohorts have included thousands of women and hundreds of endometrial cancer cases to achieve adequate precision (7). Our study of 24 patients is orders of magnitude smaller and cannot provide meaningful estimates of rare-outcome rates.

#### 4.3.2 Selection bias and missing data

##### 4.3.2.1 Exclusion of patients with unknown HRT status

Of the original 62 patients who underwent hysteroscopy, 38 (61.3%) had unknown or inadequately documented HRT status and were excluded from this analysis. This high rate of missing exposure data introduces substantial selection bias, as patients with documented HRT status may differ systematically from those without documentation in ways that affect endometrial pathology risk.

##### 4.3.2.2 Potential sources of selection bias include

Patients with more complex medical histories or higher-risk profiles may have more complete documentation; patients under active HRT management may be more likely to have HRT status documented; patients with abnormal findings may trigger more thorough chart review and documentation; and conversely, patients with routine findings may have less detailed documentation.

##### 4.3.2.3 Impact on generalizability

The restriction to patients with documented HRT status limits the generalizability of findings to the broader population of women undergoing hysteroscopy. The 24 patients included in this analysis represent only 38.7% of the original cohort and may not be representative of typical clinical practice.

##### 4.3.2.4 Incomplete HRT characterization

Even among the 24 patients with documented HRT status, detailed information about HRT regimens was often incomplete. Specific formulations were documented for only 7 of 13 HRT users (53.8%), duration of use was documented for only 9 of 13 (69.2%), and exact doses were rarely recorded. This incomplete characterization limits our ability to perform subgroup analyses by regimen type and prevents meaningful comparison with studies that have detailed exposure data.

#### 4.3.3 Retrospective design and confounding

##### 4.3.3.1 Inherent limitations of retrospective design

As a retrospective observational study, this analysis is subject to confounding by indication, unmeasured confounders, and the inability to establish temporal relationships. Women who receive HRT differ from non-users in multiple ways (e.g., symptom severity, health-seeking behavior, comorbidities, and BMI) that may independently affect endometrial pathology risk.

##### 4.3.3.2 Confounding by indication

Women prescribed HRT may have different baseline endometrial cancer risk profiles than non-users. For example, women with contraindications to HRT (such as a history of endometrial cancer or unexplained vaginal bleeding) would be systematically excluded from HRT use, potentially creating a “healthy user” bias.

##### 4.3.3.3 Unmeasured confounders

Important potential confounders were not available or incompletely documented in this dataset: BMI was available for only 1 of 24 patients (4.2%), despite BMI being a strong independent risk factor for endometrial cancer and an effect modifier of HRT-associated risk (7); diabetes status, polycystic ovary syndrome, and other metabolic conditions were not systematically documented; family history of endometrial or other cancers was not available; and detailed reproductive history beyond parity was not available.

##### 4.3.3.4 Inability to establish causation

The retrospective cross-sectional design precludes any causal inference about the relationship between HRT and endometrial histology. We cannot determine whether HRT use preceded or followed endometrial changes, nor can we account for changes in HRT regimens over time.

#### 4.3.4 Outcome ascertainment and measurement

##### 4.3.4.1 Indication bias for hysteroscopy

All patients in this study underwent hysteroscopy for clinical indications (primarily bleeding symptoms or surveillance). This introduces indication bias, as the study population is not representative of all HRT users or all postmenopausal women, but rather a selected group with symptoms or clinical concerns warranting invasive evaluation.

##### 4.3.4.2 Heterogeneity of clinical presentations

The “other” category of clinical presentations comprised 45.8% of patients, representing a heterogeneous group that may include routine surveillance, incidental findings, or non-bleeding symptoms. This heterogeneity limits the interpretability of findings and comparability with other studies.

##### 4.3.4.3 Histological classification

Histological findings were abstracted from clinical reports and categorized post-hoc. The “other” category of histological findings (25.0% of patients) represents a heterogeneous group that may include various benign findings. More granular classification was not possible due to limitations of the source data.

##### 4.3.4.4 Inadequate samples

Three patients (12.5%) had inadequate endometrial samples, representing a potential source of outcome misclassification. Inadequate sampling may be more common in certain patient groups (e.g., atrophic endometrium in non-HRT users) and could bias comparisons between groups.

#### 4.3.5 Generalizability

##### 4.3.5.1 Single-center study

Data were collected from a single institution (Princess Royal University Hospital), limiting generalizability to other healthcare settings, geographic regions, or patient populations. Prescribing patterns, patient demographics, and clinical practices may differ substantially across institutions.

##### 4.3.5.2 Contemporary UK practice

This study reflects contemporary UK clinical practice and HRT prescribing patterns, which may differ from those in other countries or healthcare systems. For example, the predominance of transdermal estradiol (38.5% of HRT users) reflects UK prescribing preferences but may not be representative of global practice.

##### 4.3.5.3 Selected population

Patients undergoing hysteroscopy represent a selected population with clinical indications for invasive evaluation and may not be representative of all HRT users or all postmenopausal women.

### 4.4 Strengths

Despite the significant limitations outlined above, this study has several strengths:

#### 4.4.1 Focus on documented cases

By restricting the analysis to patients with explicitly documented HRT status, we prioritized data quality and minimized exposure misclassification, even at the cost of reduced sample size.

#### 4.4.2 Detailed HRT characterization

We attempted to characterize HRT regimens in detail, such as type, route, schedule, and duration, providing more granular information than many retrospective studies.

#### 4.4.3 Transparent reporting of limitations

We explicitly quantified statistical power and sample size requirements, providing transparent communication of the study’s limitations and appropriate context for interpreting null findings.

#### 4.4.4 Real-world clinical data

This study provides real-world data on endometrial histology in women undergoing hysteroscopy in contemporary UK clinical practice, complementing data from randomized trials and large cohort studies.

### 4.5 Clinical implications

Given the severe methodological limitations of this small case series, the clinical implications are limited:

1. This study does not provide evidence for or against HRT safety: the absence of endometrial hyperplasia or cancer in 24 patients is consistent with low baseline incidence rates but does not demonstrate safety or lack of risk.
2. Endometrial surveillance remains important: women on HRT, particularly those with unscheduled bleeding, require appropriate endometrial evaluation according to established clinical guidelines, regardless of the findings of this small study.
3. Prescribing HRT should follow evidence-based guidelines: clinical decisions about prescribing HRT and endometrial surveillance should be based on large-scale randomized trials and cohort studies (2, 4, 7) and not on small retrospective case series.
4. Documentation of HRT regimens is important: the high rate of missing or incomplete HRT documentation (61.3% of patients with unknown status) highlights the need for improved systematic documentation of HRT type, route, dose, and duration in clinical records to facilitate future research and quality improvement.

### 4.6 Future research directions

This study highlights several important directions for future research.

#### 4.6.1 Adequately powered prospective studies

Future studies should be prospectively designed with sample sizes calculated to achieve adequate power to detect clinically meaningful differences in rare outcomes. Based on our power calculations, studies aiming to detect a 10% absolute difference in abnormal histology rates would require approximately 800 patients.

#### 4.6.2 Detailed HRT regimen characterization

Future studies should systematically collect detailed information on HRT formulations, such as specific estrogen and progestogen types and doses; route of administration (oral, transdermal, or vaginal); regimen schedule (continuous vs. sequential, days of progestogen per month); duration of use and cumulative exposure; and changes in regimens over time.

#### 4.6.3 Measurement of important confounders

Future studies should systematically measure and adjust for important confounders, such as BMI, diabetes, polycystic ovary syndrome, family history, and other metabolic and reproductive factors.

#### 4.6.4 Long-term follow-up

Endometrial cancer risk may increase with longer duration of HRT use (7). Future studies should include long-term follow-up (≥10 years) to assess the cumulative effects of HRT on endometrial outcomes.

#### 4.6.5 Subgroup analyses by regimen type

Adequately powered studies should perform pre-specified subgroup analyses comparing endometrial outcomes across different HRT regimens (estrogen-only, continuous combined, and sequential combined) and formulations (synthetic vs. micronized progestins and oral vs. transdermal estrogen).

#### 4.6.6 Integration with molecular and imaging biomarkers

Future studies could integrate histological findings with molecular biomarkers (e.g., hormone receptor expression and proliferation markers) and advanced imaging techniques to better understand mechanisms of HRT effects on the endometrium.

## 5 Conclusion

This retrospective case series of 24 women with documented HRT status undergoing hysteroscopy found no cases of endometrial hyperplasia or carcinoma in either HRT users or non-users. However, *post-hoc* power analysis revealed that this study had only 14% power to detect a 10% absolute difference in abnormal histology rates and would require 788 patients to achieve adequate power. The absence of pathological findings likely reflects the small sample size and low baseline incidence of these rare outcomes rather than a true protective effect or lack of association.

This study was severely underpowered to detect rare but clinically important endometrial pathology and should not be interpreted as providing evidence for or against HRT safety. The findings underscore the critical importance of adequate sample size and statistical power in studies of rare outcomes and highlight the limitations of small retrospective case series for informing clinical practice.

The high rate of missing HRT documentation (61.3% of the original cohort) and incomplete characterization of HRT regimens among documented users represent significant methodological limitations that constrain interpretation and generalizability. Future research should prioritize prospectively designed studies with adequate sample sizes, detailed HRT regimen characterization, systematic measurement of confounders, and long-term follow-up to inform evidence-based endometrial surveillance strategies in women receiving hormone replacement therapy.

Clinical decisions about prescribing HRT and endometrial surveillance should continue to be based on large-scale randomized trials and prospective cohort studies rather than small retrospective case series.

## Data Availability

The original contributions presented in the study are included in the article/supplementary material, further inquiries can be directed to the corresponding author.
